# A Phase 1 dose-escalation study of disulfiram and copper gluconate in patients with advanced solid tumors involving the liver using S-glutathionylation as a biomarker

**DOI:** 10.1186/s12885-021-08242-4

**Published:** 2021-05-07

**Authors:** Kristen C. Kelley, Kenneth F. Grossman, Mary Brittain-Blankenship, Kelli M. Thorne, Wallace L. Akerley, Moises C. Terrazas, Ken M. Kosak, Kenneth M. Boucher, Saundra S. Buys, Kimberly A. McGregor, Theresa L. Werner, Neeraj Agarwal, John R. Weis, Sunil Sharma, John H. Ward, Thomas P. Kennedy, Douglas W. Sborov, Paul J. Shami

**Affiliations:** 1 Department of Internal Medicine, University of Utah, Salt Lake City, Utah USA; 2 Division of Medical Oncology, Department of Internal Medicine, Huntsman Cancer Institute, University of Utah, Salt Lake City, Utah USA; 3 Huntsman Cancer Institute, University of Utah, Salt Lake City, Utah USA; 4 Division of Hematology and Hematologic Malignancies, Department of Internal Medicine, Huntsman Cancer Institute, University of Utah, 2000 Circle of Hope, Salt Lake City, Utah USA; 5 Pulmonary Diseases, Critical Care and Environmental Medicine, Tulane University, New Orleans, USA

**Keywords:** Disulfiram, Copper gluconate, *S*-glutathionylation

## Abstract

**Background:**

Disulfiram and metals inactivate key oncoproteins resulting in anti-neoplastic activity. The goal of this study was to determine the maximum tolerated dose of copper when administered with disulfiram in patients with advanced solid tumors and liver involvement.

**Methods:**

Disulfiram 250 mg was administered daily in 28-day cycles. Four doses of copper gluconate were tested (2, 4, 6, and 8 mg of elemental copper) in a standard 3 + 3 dose escalation design. Patients were evaluated for dose limiting toxicities and response. Protein *S*-glutathionylation was evaluated as a pharmacodynamic marker.

**Results:**

Twenty-one patients were enrolled and 16 patients were evaluable for dose limiting toxicities. Among the 21 patients, there was a median of 4 lines of prior chemotherapy. Five Grade 3 toxicities were observed (anorexia, elevated aspartate aminotransferase or AST, elevated alkaline phosphatase, fever, and fatigue). Response data was available for 15 patients. Four patients had stable disease with the longest duration of disease control being 116 days. The median duration of treatment for evaluable patients was 55 days (range 28–124). Reasons for discontinuation included functional decline, disease progression, and disease-associated death. Increased *S*-glutathionylation of serum proteins was observed with treatment.

**Conclusion:**

Disulfiram 250 mg daily with copper gluconate (8 mg of elemental copper) was well-tolerated in patients with solid tumors involving the liver and was not associated with dose limiting toxicities. While temporary disease stabilization was noted in some patients, no objective responses were observed. Treatment was associated with an increase in *S*-glutathionylation suggesting that this combination could exert a suppressive effect on cellular growth and protein function.

**Trial registration:**

NCT00742911, first posted 28/08/2008.

**Supplementary Information:**

The online version contains supplementary material available at 10.1186/s12885-021-08242-4.

## Background

Disulfiram is a drug best known for the treatment of alcoholism. Its anti-cancer properties were first reported in the early 1960s [1]. As a lipophilic dithiocarbamate, disulfiram crosses the cell membrane and complexes with metal ions [2]. These complexes disrupt vital signaling pathways through the formation of reactive oxygen species [3–10], interference with DNA expression [8, 11–14], anti-proteasome activity [15–22], anti-angiogenesis properties [10, 23, 24], and disruption of mitochondrial membrane permeability and polarization [25, 26]. Apoptosis in cancer cells is induced [14, 16, 20–22, 27–29] via activation of ERK and JNK pathways and p38 stress-activated protein kinases [8, 30, 31]. Copper supplementation of growth media increases pro-apoptotic [29] and anti-proteosome [16] activities of disulfiram to a greater extent than other metals.

Disulfiram-metal complexes can also re-sensitize tumor cells to chemotherapy by inhibiting the multidrug resistance P-glycoprotein [32–34] or acetaldehyde dehydrogenase, which can be increased in cancer cells [35]. Re-sensitization to 5-fluorouracil [36], cisplatin [37], gemcitabine [38], doxorubicin [39], and temozolomide [40] has been observed along with possible potentiation of cytotoxicity [38, 40, 41].

This Phase 1 trial was designed to determine the safety of disulfiram when combined with copper gluconate in treating patients with refractory malignancies metastatic to the liver. Early phase clinical trials of disulfiram-metal combinations have demonstrated a favorable safety profile across a variety of doses and schedules (Supplementary Table 1) [42–46]. The liver is a common site of metastasis for a variety of solid tumors, most commonly adenocarcinomas of the colon, pancreas, or breast [47]. Hepatic metastases portend a poor prognosis and there is a need for novel therapies to improve outcomes in this population. Hepatic antineoplastic activity is supported by a report of a patient with stage IV ocular melanoma with hepatic metastases who obtained a clinical remission for 53 months after treatment with zinc gluconate and disulfiram [11].

## Methods

### Patient selection

Patients 18 years of age or older with solid tumors, hepatic metastases, and an expected survival of at least 3 months were eligible. Patients were to have exhausted or refused standard therapies for their disease. Patients had to refrain from alcoholic beverages while on study and could not be receiving other chemotherapy while enrolled. Baseline aspartate aminotransferase (AST) and alanine transaminase (ALT) levels less than 5 times the upper limit of normal, normal serum copper levels, and serum ceruloplasmin greater than 17 mg/dL were required. Of note, the trial was amended to change the eligibility criteria for baseline AST and ALT from less than 2.5 times the upper limit of normal to less than 5 times the upper limit of normal. With this being a trial that involves liver metastasis, it is expected that patients will have elevated liver enzymes in this setting. As a result of the change in eligibility, the dose limiting toxicity (DLT) criteria were amended at the same time to exclude Grade 3 or 4 liver function abnormalities as DLT if the patient had Grade 3 or 4 liver function abnormalities at baseline. These patients were closely monitored to see if any trend could be seen relating to study drug administration. This amendment did not impact any previous patient enrollments.

Exclusion criteria included active liver disease other than metastatic cancer, Eastern Cooperative Oncology Group (ECOG) performance status of 3 or 4, pregnant or nursing women, and women of childbearing potential who were not using contraception. Patients with a family history of Wilson’s disease or hemochromatosis were excluded as were those taking medications metabolized by cytochrome P450 2E1 or those whose metabolism is likely influenced by disulfiram.

### Study design and treatment

Patients were enrolled between 2008 and 2011 at the University of Utah Huntsman Cancer Institute. The study was conducted under Investigational New Drug (IND) #100,937 and approved by the University of Utah Institutional Review Board. The study was designed as a standard 3 + 3 dose escalation to determine the maximum tolerated dose (MTD) or maximum administered dose (MAD) of copper gluconate with a fixed dose of disulfiram. Toxicities were Graded according to the Cancer Therapy Evaluation Program Common Terminology Criteria for Adverse Events Version 3. To be evaluable for dose-limiting toxicity (DLT) assessment, patients were required to complete 28 days of treatment. A DLT was defined as Grade 3 or higher nausea, persistent Grade 4 neutropenia, febrile neutropenia, Grade 4 thrombocytopenia or Grade 3 thrombocytopenia with bleeding or transfusion needs, Grade 4 anemia, or any Grade 4 non-hematologic toxicity.

Patients were assigned to one of 4 dose cohorts of copper gluconate with an 8 mg maximum dose of elemental copper (Table 1) together with disulfiram at a fixed dose of 250 mg orally per day. Disulfiram was prescribed from commercial sources. Copper gluconate was obtained from Twin Labs (American Fork, UT). Disulfiram was taken with the evening meal separate from copper gluconate in the morning to minimize gastrointestinal toxicity. 28-day treatment cycles continued until toxicity or disease progression. All patients, including those who discontinue protocol therapy early, were followed for response until progression and for survival for up to 2 years from the date of registration.

**Table 1:** Dose escalation scheme

Cohort	Copper Gluconate	Disulfiram
**1**	**2 mg**^a^	**250 mg**
**2**	**4 mg**	
**3**	**6 mg**	
**4**	**8 mg**	

^a^*mg* milligrams of elemental copper

### Outcomes

The primary outcome was the safety, tolerability, and MTD of disulfiram and copper gluconate in patients with hepatic metastases. Secondary outcomes included treatment response using RECIST v.1.0 criteria (data analysis occurred prior to RECIST v. 1.1) and a qualitative assessment of the induction of *S*-glutathionylation in proteins of circulating leukocytes. Treatment response was assessed with measurement of tumor markers with each cycle and CT scans of the chest, abdomen, and pelvis which were obtained after every two cycles of treatment to evaluate response.

### Statistical analysis

Statistical analysis was descriptive as the power of a 3 + 3 study to accurately determine the real rate of toxicity at a given dose is limited. Response rates and correlative studies likewise involved small sample numbers of different malignancies and thus these outcome measures are also descriptive in nature.

### Pharmacodynamics

We hypothesized that this combination would inactivate proteins important for malignant cell growth through *S-*glutathionylation of cysteine residues on proteins. *S*-glutathionylation assays were performed on plasma proteins at baseline and on day 8 of treatment. Plasma proteins were isolated from the peripheral blood and 30 mcg of protein were separated by SDS-PAGE under non-reducing conditions. Following transfer, *S-*glutathionylation levels were evaluated using Western blotting procedures with primary anti-glutathione mouse monoclonal antibody (ViroGen, Watertown, MA). Loading control was performed with a mouse anti-human IgG (Sigma-Aldrich, St. Louis, MO).

## Results

### Patients

Twenty-one patients were enrolled and treated. Sixteen were evaluable for DLTs. Of those, 11 were women. Thirteen patients were Caucasian, two were Native American/Alaska Native, and 1 was Asian. Five patients were not evaluable because they received less than 28 days of treatment (Supplementary Table 2). The median age of the evaluable patients was 58 (range 27–81). Tumor types included pancreatic adenocarcinoma, colorectal adenocarcinoma, non-small cell lung carcinoma, cutaneous and ocular melanoma, thymic carcinoma, and breast carcinoma. The median number of prior lines of treatment was 4 (range 0–12) with 11 of the 16 evaluable patients also having received prior radiation (Table 2).

**Table 2:** Characteristics of evaluable patients

ID	Cohort	Age	Sex	Race^**a**^	Primary Tumor Type	Prior Treatments^**b**^
**1**	**1**	**63**	**M**	**NA**	**Pancreas**	**3**
**2**		**34**	**M**	**NA**	**Colon**	**6**
**3**		**62**	**F**	**C**	**Lung**	**5**
**4**	**2**	**57**	**F**	**C**	**Melanoma (Ocular)**	**1**
**5**		**27**	**M**	**C**	**Thymic**	**3**
**6**		**44**	**F**	**C**	**Breast**	**12**
**7**	**3**	**66**	**F**	**C**	**Melanoma (Cutaneous)**	**1**
**8**		**70**	**F**	**C**	**Melanoma (Cutaneous)**	**1**
**9**		**70**	**M**	**C**	**Melanoma (Cutaneous)**	**1**
**10**	**4**	**43**	**F**	**C**	**Breast**	**7**
**11**		**59**	**F**	**C**	**Melanoma (Ocular)**	**0**
**12**		**57**	**M**	**C**	**Melanoma (Cutaneous)**	**5**
**13**		**81**	**F**	**C**	**Melanoma (Ocular)**	**0**
**14**		**57**	**F**	**C**	**Breast**	**6**
**15**		**61**	**F**	**A**	**Breast**	**9**
**16**		**53**	**F**	**C**	**Breast**	**6**

^a^ *C* Caucasian, *NA* Native American or Alaska Native, *A* Asian

^b^ Including chemotherapy and endocrine therapy. *ID* identification, *M* male, *F* female

### Safety

No DLTs were observed. Overall, there were 53 adverse events felt to be possibly, probably, or definitely related to study treatment in the evaluable patients. These are summarized in Table 3. The most common adverse events were abnormalities in liver function tests, fatigue, nausea, dysgeusia, and vomiting. The majority of events (*n* = 30) occurred in Cohort 4 including three of the 5 Grade 3 events. These included elevated AST and alkaline phosphatase, fatigue, anorexia, and fever. There were no Grade 4 or 5 events attributed to study treatment.

**Table 3:** Common toxicities attributable to study treatment in evaluable patients

Event	Grade 1	Grade 2	Grade 3	Total
**Elevated AST**	**3**	**2**	**1**	**6**
**Fatigue**	**4**	**1**	**1**	**6**
**Elevated ALT**	**3**	**1**	**0**	**4**
**Nausea**	**3**	**1**	**0**	**4**
**Taste changes**	**2**	**1**	**0**	**3**
**Vomiting**	**2**	**1**	**0**	**3**
**Dizziness**	**3**	**0**	**0**	**3**
**Elevated alkaline phosphatase**	**1**	**1**	**1**	**3**
**Anorexia**	**1**	**0**	**1**	**2**
**Hyperbilirubinemia**	**1**	**1**	**0**	**2**
**Thrombocytopenia**	**1**	**1**	**0**	**2**
**Hypocalcemia**	**1**	**0**	**0**	**1**
**Hypokalemia**	**1**	**0**	**0**	**1**
**Hyponatremia**	**1**	**0**	**0**	**1**
**Hypoalbuminemia**	**1**	**0**	**0**	**1**
**Constipation**	**1**	**0**	**0**	**1**
**Flatulence**	**1**	**0**	**0**	**1**
**Cough**	**1**	**0**	**0**	**1**
**Fever**	**0**	**0**	**1**	**1**
**Headache**	**1**	**0**	**0**	**1**
**Hypertension**	**1**	**0**	**0**	**1**
**Memory impairment**	**1**	**0**	**0**	**1**
**Pain (leg)**	**1**	**0**	**0**	**1**

In Cohort 1, three of the 7 patients enrolled were evaluable. Reasons for lack of evaluation included medication non-adherence in 1 patient and rapid disease progression in 3 patients.

Three patients in Cohort 2 were evaluable. Two Grade 3 adverse events were recorded that were possibly related to the study treatment and included elevated alkaline phosphatase and AST. The Grade 3 alkaline phosphatase elevation event occurred 1 month after treatment initiation in a patient with extensive hepatic involvement. This improved after discontinuation; however, this may have reflected a treatment response as the patient also had decreasing tumor markers with the next line of therapy.

Three of the 4 patients in Cohort 3 were evaluable. No Grade 3 or higher events occurred within this cohort. One patient was not evaluable due to death related to disease progression 18 days after starting treatment.

Seven patients were enrolled in Cohort 4. One patient experienced Grade 3 fatigue and anorexia. This patient was also noted to have Grade 2 dysgeusia that likely contributed to decreased oral intake. These side effects resolved within 2 weeks of treatment discontinuation for disease progression. Grade 3 fever was experienced by one patient in this Cohort. In addition to the above, reversible and low-Grade neurologic side effects were observed including memory impairment (*n* = 1) and headache (n = 1) in Cohort 1, and dizziness (*n* = 3) and auditory changes (n = 1) in Cohort 4.

There were five Grade 1–2 adverse events recorded among non-evaluable patients including a Grade 2 allergic reaction to disulfiram occurring in Cohort 1. All other events occurred in Cohort 2.

### Efficacy

Response data was available for 15 of the 16 evaluable subjects (Table 4). Patient 9 died prior to first imaging assessment. Eleven patients had disease progression and 4 had stable disease (mean 96 days, range 42–124). One patient in Cohort 3 was characterized as having symptomatic progression without evidence of progression per RECIST and was categorized as having stable disease. No partial or complete responses were observed. One heavily pretreated patient with colorectal cancer had disease stabilization for 124 days in Cohort 1.

**Table 4:** Best Response in all evaluable patients

ID	Cohort	Best Response^**a**^	Days on Study
**1**	**1**	**PD**	**43**
**2**		**SD**	**124**
**3**		**PD**	**32**
**4**	**2**	**PD**	**40**
**5**		**PD**	**55**
**6**		**PD**	**26**
**7**	**3**	**SD**	**42**
**8**		**PD**	**56**
**10**	**4**	**PD**	**56**
**11**		**SD**	**109**
**12**		**PD**	**28**
**13**		**SD**	**109**
**14**		**PD**	**34**
**15**		**PD**	**43**
**16**		**PD**	**56**

^a^*PD* progressive disease, *NA* not available

### Pharmacodynamics

**Fig. 1:**
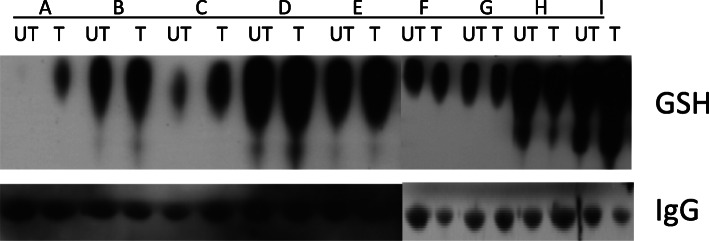
Western blot assays were performed on serum sampled from patients prior to treatment, and at one-week post treatment. UT = untreated, T = treated. White space delineates cropping of original gel image for clarity, all important bands retained

Pharmacodynamic data were available for 9 patients. Increased serum protein *S-*glutathionylation was observed in 6 patients (Fig. 1). Two of the patients had no change in serum protein *S-*glutathionylation and one patient had a decrease.

## Discussion

Daily disulfiram at 250 mg with a maximum 8 mg of elemental copper was well-tolerated with no DLTs. Elevation of liver function tests may have been treatment-related. However, this population was selected for hepatic metastases. Neurologic side effects were of low grade and reversible though more common with higher doses of copper. A decline in performance status due to other factors such as anorexia, disease progression, or concomitant central nervous system disease in some patients make the exact attribution of these neurologic adverse events difficult in this small study.

Different cancers are associated with increased intratumoral copper levels or altered systemic copper distribution [48]. Copper promotes tumor progression by playing critical roles in angiogenesis and metastasis [48]. Consequently, copper-chelating agents (such as tetrathiomolibdate) have been proposed for the treatment of malignancies. However, it has been suggested that copper chelation alone is insufficient to kill malignant cells and that for cancer treatment, chelating agents should be combined with other drugs [48]. On the other hand, disulfiram-metal complexes show promising anti-cancer activity both in preclinical models and early phase clinical trials. The limited data currently available demonstrate mixed results in regard to efficacy, though an increase in survival was observed in a study of disulfiram and chemotherapy in non-small cell lung cancer [46]. Data suggest that disulfiram alone or in combination with chemotherapy or metals is safe and well-tolerated with disulfiram dosing ranging from 40 mg three times daily to 2000 mg/m^2^.

In our study no partial or complete responses were seen. This may be due in part to inadequate drug concentrations with once daily dosing, or the fact that we enrolled heavily pretreated patients, or the small sample size of our study. Patients with solid tumors or those with metastases may not be ideal responders to disulfiram-metal combinations. Combining disulfiram-metal treatment with cytotoxic chemotherapy may yield synergistic responses and remains a promising area of investigation.

There is a clear need for predictive biomarkers. Deletion p16 in B-cell ALL cells and in patient-derived xenografts may predict efficacy of disulfiram and copper [49]. Similarly, BRCA-deficient cells have demonstrated increased responses to disulfiram in in vitro models due to increased susceptibility to acetaldehyde toxicity [50]. Loss of 16q, a genetic abnormality observed in many tumor types, may increase the bioavailability of copper and therefore the potency of treatment [51].

In their study of the combination of disulfiram and zinc, Brar et al. have suggested that dithiocarbamate/metal complexes disrupt transcription factor binding to DNA by inducing *S*-glutathionylation [11]. In addition, Paranjpe et al. have shown that disulfiram, with or without copper, induces degradation of NF-κB, and induces *S*-glutathionylation and degradation of p53 [52]. Degradation of p53 and NF-κB correlates with decreases in their specific binding to DNA, suggesting that *S*-glutathionylation inhibits the functional activity of the proteins. We therefore hypothesized that the combination of disulfiram and copper could induce protein *S-*glutathionylation *in vivo*. Our pharmacodynamics data show that *S-*glutathionylation of serum proteins tends to increase following treatment with disulfiram and copper. In *S-*glutathionylation, glutathione is reversibly conjugated to free thiols on protein cysteine residues forming mixed disulfide bonds [53] with resultant inhibition of protein function. Disulfiram induces *S*-glutathionylation and inactivation of proteins important for cell survival [11] including Jun, NF-κB, ATF/CREB, and other proteins involved in cell proliferation [54, 55]. *S*-glutathionylation inhibits glycolysis [56] and thus may exert a negative effect on tumor metabolism. While *S*-glutathionylation of serum proteins does not necessarily imply protein *S*-glutathionylation in tumor cells, our findings suggest that such a mechanism may contribute to the antineoplastic activity of the combination. Our sample size was too small to allow correlation between *S-*glutathionylation and response. However, this will be important to investigate in future larger trials.

## Conclusions

This Phase 1 trial of disulfiram in combination with up to 8 mg of elemental copper demonstrated safety and tolerability in patients with metastatic solid tumors to the liver. While some patients had stable disease, no objective responses were observed. Given likely non-overlapping toxicities and potential for synergy, use of disulfiram-metal combinations with cytotoxic chemotherapy is an attractive avenue of investigation which enhance efficacy. As supported by promising emerging preclinical data, application of this therapy to other disease states including hematologic malignancies in a biomarker-driven fashion may result in more significant clinical benefit [51]. The excellent safety profile of this combination together with the presence of multiple identified therapeutic targets, ease of use, drug availability, and low-cost warrant further study of disulfiram and metals in the treatment of cancer.

## Supplementary Information

**Additional file 1: Supplementary Table 1.** Clinical trials investigating disulfiram in patients with solid tumors. **Supplementary Table 2.** Characteristics of non-evaluable patients.

## Data Availability

The datasets used and/or analyzed during the current study are available from the corresponding author on reasonable request.

## Abbreviations

ALT: Alanine transaminase
AST: Aspartate aminotransferase
DLT: Dose-limiting toxicity
ECOG: Eastern Cooperative Oncology Group
IND: Investigational new drug
MAD: Maximum administered dose
MTD: Maximum tolerated dose
