# Supplementary material for: A Phase 1 dose-escalation study of disulfiram and copper gluconate in patients with advanced solid tumors involving the liver using S-glutathionylation as a biomarker
Source: BMC Cancer. 2021 May 7;21:510. doi: 10.1186/s12885-021-08242-4 (PMC8103752; doi:10.1186/s12885-021-08242-4)
Supplement: Supplementary file 1 — Additional file 1: Supplementary Table 1. Clinical trials investigating disulfiram in patients with solid tumors. Supplementary Table 2. Characteristics of non-evaluable patients. [file 12885_2021_8242_MOESM1_ESM.docx]

**Title:**  A phase 1 dose-escalation study of disulfiram and copper gluconate in patients with advanced solid tumors involving the liver using S-glutathionylation as a biomarker

**Authors:** Kristen C. Kelley, MD^1^; Kenneth F. Grossman, MD, PhD^2^; Mary Brittain-Blankenship, MD^1^; Kelli M. Thorne^3^, MS; Wallace L. Akerley^2^, MD; Moises C. Terrazas, MS^4^; Ken M. Kosak^4^, Kenneth M. Boucher, PhD^1^; Saundra S. Buys, MD^2^; Kimberly A. McGregor, MD^2^, Theresa L. Werner, MD^2^; Neeraj Agarwal, MD^2^; John R. Weis, MD^2^; Sunil Sharma, MD, FACP, MBA^2^; John H. Ward, MD^2^; Thomas P. Kennedy, MD^5^; Douglas W. Sborov^4^, MD, MS; Paul J. Shami, MD^4^

**Affiliations:**

^1^Department of Internal Medicine, University of Utah

^2^Division of Medical Oncology, Huntsman Cancer Institute, University of Utah

^3^Huntsman Cancer Institute, University of Utah

^4^Division of Hematology and Hematologic Malignancies, Huntsman Cancer Institute, University of Utah

^5^Pulmonary Diseases, Critical Care and Environmental Medicine, Tulane University

KAG, TPK, and SS are no longer affiliated with Huntsman Cancer Institute. KAJ is affiliated with Foundation Medicine, TPK with Tulane University School of Medicine, and SS with the Translational Genomics Research Institute (TGen).

**Correspondence**:

Paul J. Shami, MD

Huntsman Cancer Institute

2000 Circle of Hope, Suite 2100

Salt Lake City, UT 84112-5550

Paul.shami@utah.edu, 801-585-5136

**Supplementary Table 1. Clinical trials investigating disulfiram in patients with solid tumors**

| **Trial Author** | **Treatment^a^** | **Disease^b^** | **N** | **Design^c^** | **Dosing^d^** | **Safety notes^e^** | **Efficacy^f^** |
| --- | --- | --- | --- | --- | --- | --- | --- |
| **Huang^42^** | **DSF-Cu + TMZ** | **Recurrent GBM (TMZ-resistant)** | **23** | **P2 single arm** | **DSF: 80 mg tid**  **Cu: 1.5 mg tid** | **One DLT = grade 3 elevated ALT** | **14% clinical benefit**  **PFS 1.7 m, OS 7.1 m** |
| **Schweizer^43^** | **DSF** | **Recurrent localized prostate cancer** | **19** | **P1 dose escalation** | **DSF: 250 mg daily (n=9), 500 mg (n=10)** | **Six patients experienced grade 3 AEs (3 per cohort)** | **NA** |
| **Grossman^44^** | **DSF + Zn** | **Metastatic melanoma** | **12** | **P2 single arm** | **DSF: 250 mg qhs**  **Chelated Zn: 50 mg tid** | **One grade 3 AE = confusion** | **PFS 53 d, OS 203 d** |
| **Verma^45^** | **DSF + cis** | **Cisplatin-sensitive malignancies** | **53** | **rP2 Chemo +/- DSF** | **Cis: 100 mg/m2**  **DSF: 2000 mg/m2** | **Addition of DSF group increased grade of GI and ototoxicity** | **No difference in response, TTP, or OS** |
| **Nechushtan^46^** | **DSF + cis + vin** | **NSCLC** | **40** | **rP2 Chemo +/- DSF** | **DSF: 40 mg TID** | **NA** | **DSF improved PFS (5.9 vs 4.9 m) and OS (10 vs 7.1 m)** |

^a^DSF = disulfiram, cis = cisplatin, vin = vinorelbine, Cu = copper, TMZ = temozolomide, Zn = zinc ^b^NSCLC = non-small cell lung cancer, GBM = glioblastoma multiforme ^c^r = randomized, P = phase, Chemo = chemotherapy ^d^tid = three times daily, n = number, mg = milligram, m = meter, qhs = nightly, NA = not available ^e^DLT = dose limiting toxicities, AE = adverse event ^f^SD = stable disease, m = month, PFS = progression free survival, OS = overall survival, d = day, TTP = time to progression

^f^SD = stable disease, m = month, PFS = progression free survival, OS = overall survival, d = day, TTP = time to progression

| **Dose Cohort^a^** | **Age** | **Sex^b^** | **Race^c^** | **Primary Tumor Type** | **Prior Lines of Treatment^d^** | **Reason for discontinuation^e^** |
| --- | --- | --- | --- | --- | --- | --- |
| **2 mg** | **56** | **F** | **C** | **Pancreas** | **4** | **PD** |
| **2 mg** | **51** | **F** | **C** | **Breast** | **13** | **PD** |
| **2 mg** | **62** | **F** | **C** | **Breast** | **8** | **PD** |
| **2 mg** | **66** | **M** | **C** | **Prostate** | **3** | **PD** |
| **6 mg** | **78** | **F** | **C** | **Urothelial** | **5** | **PD** |

**Supplementary Table 2. Characteristics of non-evaluable patients.**

^a^mg = milligrams ^b^F = female, M = male ^c^C = Caucasian ^d^Refers to prior chemotherapy or endocrine therapy ^e^PD = progressive disease
